# Practices, management, and typology of dromedary livestock systems and health constraints in southwestern Tunisia: The case of the Gafsa region

**DOI:** 10.1016/j.vas.2026.100646

**Published:** 2026-04-01

**Authors:** Raoudha Sadraoui, Sana Khaldi, Imen Kallel, Mohamed Jaouad

**Affiliations:** a Laboratory of Biotechnology and Bio-monitoring of the Environment and Oasis Ecosystems - Department of Life Sciences - Faculty of Sciences of Gafsa, 2112, Tunisia; b Department of Reproductive Sciences and Pathology National School of Veterinary Medicine Sidi Thabet, 2020 Tunisia; c Research Laboratory of Environmental Toxicology-Microbiology and Health (LR17ES06), Faculty of Sciences, University of Sfax, Tunisia; d Laboratory of Economics (LR16IRA05), Institute of Arid Regions – Medenine

**Keywords:** Dromedary, Production system, Typology, Tunisia, *Trypanosoma evansi*

## Abstract

Dromedary production plays a key role in the socio-economic systems of arid regions in North Africa; however, information on the organization and management of camel production systems remains limited. This study aimed to characterize dromedary breeding systems in southwestern Tunisia (Gafsa governorate) using a typological approach. From April to August, a field survey was conducted among breeders selected from an official list provided by the Office of Livestock and Pastures. A total of 43 farms were randomly selected, representing 1195 Maghrebi dromedaries, including 1026 females. Data on farm structure, herd composition, and management practices were collected using a structured questionnaire. Multivariate analysis showed that camel production systems are predominantly traditional and extensive. Four types of farms were identified: (i) large-scale camel-oriented farms (7 %) characterized by the highest abortion (39 %) and juvenile mortality (35 %) rates; (ii) mixed livestock farms combining dromedaries with small ruminants (81 %); (iii) specialized sheep breeders operating under strong land constraints (5 %); and (iv) diversified mixed-species farms integrating goats and poultry production (7 %).

Serological analysis using the CATT/T. *evansi* test revealed a high prevalence (90.91 %) of *Trypanosoma evansi* infection, indicating an important health constraint. These findings highlight the need to improve herd management and strengthen disease control strategies to enhance the sustainability of camel production systems in arid regions.

## Highlights

• First typological study of camel breeding systems in Gafsa, Southwestern Tunisia.
• High prevalence of Trypanosoma evansi infection (90.9 %) in surveyed herds.
• Four distinct breeder groups identified based on herd size, practices, and constraints.
• Major constraints include poor technical management, malnutrition, and high abortions.
• Findings support strategies to improve camel resilience and productivity in arid zones.

## 1 Introduction

Dromedary farming is a small but specialized and strategically important part of Tunisia's larger livestock industry. It is important for the livelihoods of people in the southern parts of the country, where dry and semi-dry conditions are best for camel husbandry (Moslah et al., 2004; Selmi et al., 2017). The dromedary (Camelus dromedarius) can provide a wide range of products, such as milk, meat, leather, hair, and manure. This makes it an important part of mixed livestock production systems (Konuspayeva et al., 2021; Sghaier et al., 2023).

In addition to its useful functions, breeding dromedaries is important for the economy and culture of pastoral families. It helps with food security, gives people more ways to make money, and makes them more resilient to changes in the weather and other environmental stressors that are becoming more common in dry areas (Faye, 2022; Sghaier et al., 2023). In addition, camels are an important part of the culture and traditions of desert communities. They are not only a source of income, but they are also a symbol of social identity and heritage.

But there are a number of problems with dromedary production systems in Tunisia. These include a lack of technical knowledge among breeders, a lack of veterinary care, and serious health problems. Surra, caused by *Trypanosoma evansi*, is one of the most common and harmful parasitic diseases in camel populations. It can cause severe symptoms like anemia, reproductive problems, abortions, and higher death rates (Faye et al., 2013; Aregawi et al., 2019). Recent studies indicate a significant prevalence of trypanosomosis in southern Tunisia, underscoring the susceptibility of extensive production systems to parasite transmission (Kalthoum et al., 2021).

These limitations lead to less-than-optimal productivity and highlight the need for a thorough description of farming methods, herd management techniques, and the problems that come with them. To make livestock production in dry and semi-arid areas more efficient and sustainable, we need to know more about the structure and variety of camel production systems.

The dromedary is very well suited to tough places like dry areas, steppes, and halophytic rangelands. Its physiological and behavioral adaptations facilitate the efficient exploitation of limited vegetation and extensive communal grazing resources. Five types of the Maghrebi dromedary have been identified in southern Tunisia, according to data from national livestock development institutions (Livestock and Pasture Office and Regional Commissions for Agricultural Development) and camel breeders (Chniter et al., 2013). According to official statistics, the Maghrebi camel is the most common breed in Tunisia and is prized for its ability to produce both meat and milk (MAEWR, 2001).

The Tunisian camel population has undergone a marked decline from 1960s to the mid-1970s, followed by a gradual recovery and stabilization in recent decades, as evidenced by FAO time-series data. Current estimates place the national herd between 60, 000 and80,000 head, with a strong spatial concentration in Southern Tunisia, where more than 80 % and up to 96 % of camels are raised within extensive pastoral systems. Camel Breeding represents a key socio-economic activity, contributing a modest share (approximately 2-4 %) to national red meat production (Jemli et al., 2018; Chehida et al., 2021). There are about 2,300 breeders in this field, (73 % south, 21 % center, 6 % north, and almost 80 % of the national herd is in southern Tunisia, which is where most of the extensive pastoral production systems are.

Even though there have been efforts in the last few decades to improve camel production in Tunisia, the industry is still mostly traditional and relies on natural forage from arid and desert rangelands in the central and southern parts of the country. Productivity stays relatively low because of a number of zootechnical, nutritional, and health problems (Faye et al., 2013). Also, the Tunisian camel sector faces significant challenges stemming from a lack of dependable quantitative data and inadequate institutional structures, leading to under-reporting and limited surveillance capabilities that hinder a thorough comprehension of herd dynamics and management strategies (Eckstein et al., 2022; Letaief & Bedhiaf‑Romdhani, 2025). Furthermore, the absence of systematic monitoring of seasonal and cross-regional livestock movements exacerbates this situation, thereby complicating the characterization of mobility patterns and the evaluation of associated disease risks (Jahel et al., 2020). Historical examinations of the Tunisian camel sector also reveal deficiencies in regulatory frameworks and professional organization, which further impede data collection and policy development (Jemli et al., 2018).

Due to these difficulties, a thorough description of dromedary farming systems is needed to help with better management and better integration of the sector, especially in the governorate of Gafsa. The aim of this study is to examine the diversity of dromedary production systems in the study region and to pinpoint the primary technical, health, and socio-economic challenges that influence their performance, with a specific focus on the prevalence and effects of *Trypanosoma evansi* infection.

## 2 Material and methods

### 2.1 Study area

**Fig. 1:**
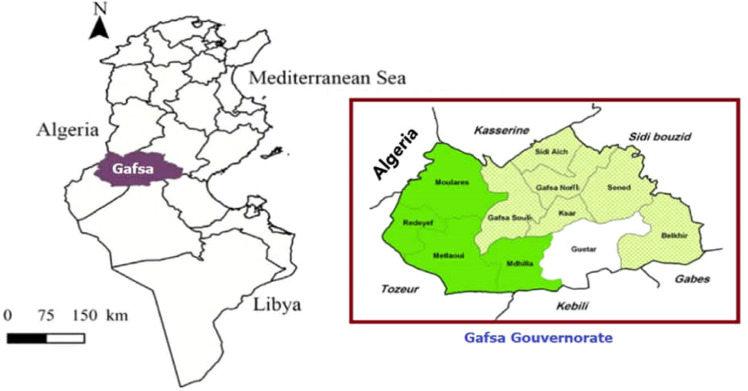
Map of the location of the Gafsa governorate, southwestern Tunisia.

The governorate of Gafsa is located in the southwest of Tunisia and constitutes a transition zone between the high and low steppes of central Tunisia. Its landscape is predominantly pre-Saharan. The region comprises 11 delegations: North Gafsa, South Gafsa, Metlaoui, Moulares, M’Dhilla, Ksar, Redeyef, El Guettar, Sidi Aïch, Sened and Belkhir (Fig. 1). Gafsa covers approximately 780,000 ha and hosts a population of about 350,394 inhabitants.

The study area lies within the arid lower Mediterranean bioclimatic zone. It is characterized by hot, dry summers and mild winters, with a mean annual temperature of 19.3 °C. Extreme temperatures can reach 49 °C in summer and −6 °C in winter. Annual rainfall is low and irregular, ranging between 150 and 180 mm, mostly occurring between September and May.

Natural resources are constrained by poor soils, recurrent droughts and pronounced interannual climatic variability, which contribute to vegetation degradation. Rangelands represent the principal feed resource and cover approximately 338,820 ha (44.3 % of the governorate). In this agro-ecological context, camelids and small ruminants are the most adapted livestock species, owing to their ability to utilize scarce and heterogeneous forage resources in arid environments.

### 2.2 Seroprevalence of *Trypanosoma evansi*

The investigation focused on dromedaries aged 12–18 months, with an average body weight of approximately 130 kg. Animals were sampled in the regions of M’Dhilla, South Gafsa, and North Gafsa as part of a coordinated blood sampling and vaccination campaign. Blood was collected by jugular venipuncture using disposable sterile equipment to prevent cross-contamination. Each sample was stored in a heparinized tube, clearly labeled with the animal’s identification. Samples were immediately placed on ice and transported to the laboratory. After centrifugation, the plasma was frozen at –20 °C until analysis. The diagnosis of *Trypanosoma evansi* was performed at the Institute of Veterinary Research of Tunisia (IRVT) using the Card Agglutination Test (CATT/*T. evansi*). This serological method, based on species-specific monoclonal antibodies, was employed to ensure high diagnostic sensitivity and specificity, allowing for the definitive identification of the parasite and the determination of its seroprevalence in the studied dromedary herds.

All animal handling and sampling procedures were carried out with strict adherence to ethical and welfare standards. The study protocol was approved by the Animal Ethics Committee of the Faculty of Sciences of Gafsa (Ethics Reference No. UG/FSG-33/25) and complied with national and international regulations governing the ethical use of animals in scientific research.

### 2.3 Typological analysis of dromedary breeding

#### 2.3.1 Data collection

A field survey was conducted among dromedary breeders located in the different delegations of the Gafsa governorate over a five-month period (April–August). The structured questionnaire used for the field survey is provided as Supplementary Material (File S1).The sample of breeders was selected from an exhaustive list of dromedary breeders provided by the Office of Livestock and Pastures (OEP), which served as the sampling frame for the study. From this list, 43 breeders were randomly selected across the governorate, ensuring representation of the main delegations where camel farming is practiced. Data were collected using a structured questionnaire specifically designed for this study and administered during farm visits carried out by the research team, occasionally in collaboration with technicians from the Office of Livestock and Pastures. Access to certain farms was facilitated by the ongoing blood-sampling and vaccination campaign conducted in the region during the survey period.

In total, 43 private farms were surveyed, representing 1195 dromedaries, including 1026 females. The questionnaire collected information on both the structural characteristics and socio-economic profiles of the farms. Variables included the farmer’s age, education level, and main economic and agricultural activities, as well as the crop and livestock enterprises present on the farm.

Additional data were gathered on herd structure and management practices, including herd establishment history, number of breeding females, origin of animals, crossbreeding practices, culling age, feeding strategies, and reproductive management parameters such as mating practices, herd sex ratio, calving patterns, fertility, prolificacy, and abortion rates. Information on housing conditions, herding practices, and sanitary prophylaxis measures was also recorded.

### 2.4 Statistical analysis

**Fig. 2:**
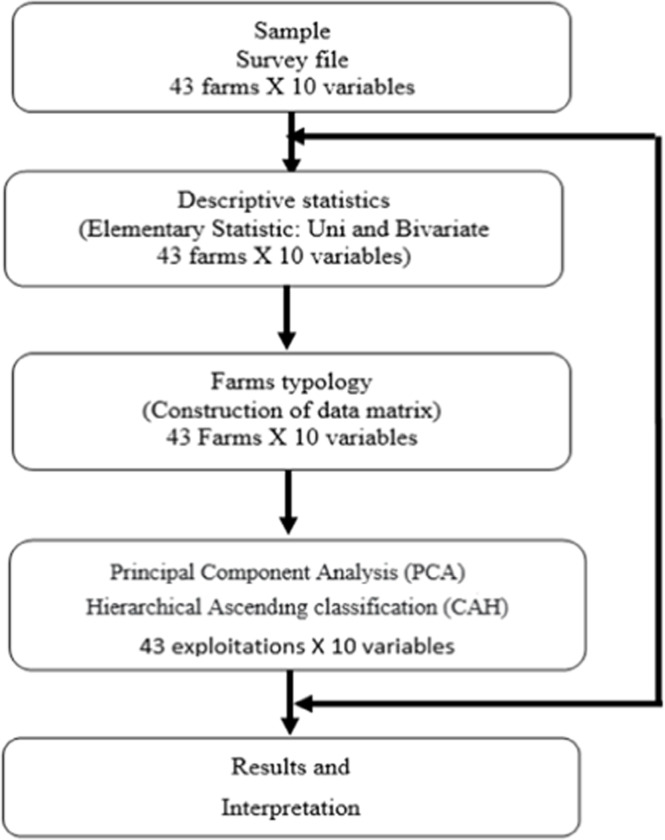
Analysis diagram of the typology of agricultural farms.

The methodological framework adopted in this study follows the typological analysis approach described by Jaouad et al. (2007), which combines descriptive statistics with multivariate analysis techniques (Fig. 2).

## 3 Descriptive analysis

Descriptive analysis constituted the first stage of data processing and aimed to ensure the quality, consistency, and interpretability of the collected data. Initially, a data validation phase was carried out to verify the accuracy, coherence, and completeness of the dataset.

Subsequently, the data were synthesized and standardized to facilitate interpretation. Descriptive statistical indicators were then calculated, including frequencies, means, standard deviations, and minimum and maximum values.

This initial analytical stage provided an overview of dromedary herd management practices and highlighted the variability of key parameters across farms. It also enabled the identification of variables showing the greatest variability among breeders, which were subsequently used in the typological and multivariate analyses.

## 4 Multivariate statistical analysis and typology

Two complementary analytical approaches were applied.

First, descriptive statistical analyses and analysis of variance (ANOVA) were performed using SAS software (SAS Institute, 1985). These analyses allowed the calculation of summary statistics (means, standard deviations, and frequencies) and the comparison of mean values among identified groups.

Second, multivariate statistical analyses were conducted using two multidimensional techniques: Principal Component Analysis (PCA) and Hierarchical Agglomerative Clustering (HAC). These methods were used to identify relationships among variables, structure the variability within the dataset, and establish a typology of dromedary breeding systems.

The analytical approach was based on Pearson correlation coefficients and followed methodological frameworks commonly applied in livestock system typology studies (Gibon, 1994; Jaouad et al., 2007).

All survey data were entered into a structured database, where each row corresponded to an individual breeder and each column represented a variable or response to a survey question. The dataset was subsequently checked for consistency and completeness, and variables were transformed and standardized when necessary to meet the assumptions required for multivariate statistical analyses. For the multivariate analyses, all individuals were retained, while a selected set of discriminant variables was used to establish the typology of breeding systems (Table 1).

**Table 1:** Description of the variables used for the Principal Component Analysis (PCA).

Variables	Code	Mean	Standard deviation
Total Farm Area (ha)	TFA	36.93	45.17
Dromedary Mortality Rate (%)	DMR	10.51	14.10
Reproductive Abortion Rate (%)	ABRT	22.84	29.11
Total Dromedary Herd Size (head)	DHERD	27.79	27.88
Number of Dromedaries Sold (head)	DSOLD	3.28	6.84
Dromedary Sale Price (TND/head)	DPRC	4,123.26	8,138.54
Number of Young Sheep (head)	YSH	37.53	63.74
Number of Adult Sheep (head)	ASH	75.28	95.08
Poultry Flock Size (head)	PFLT	13.98	20.73
Sheep Sale Price (TND/head)	SPRC	834.767	140.783

*(TND: The Tunisian Dinar), (ha: One hectare)

## 5 Results and discussion

### 5.1 Typological study of dromedary breeding

#### 5.1.1 Analysis of principal components: Choice of variables

To identify the main factors explaining variability among the surveyed camel-sheep farming systems, Principal Component Analysis (PCA) was conducted. Prior to the analysis, all quantitative variables were standardized to eliminate scale effects. The suitability of the dataset for Principal Component Analysis (PCA) was assessed using the Kaiser–Meyer–Olkin (KMO) measure and Bartlett’s test of sphericity. The KMO value (0.68) indicates an adequate level of correlation among variables, supporting the application of multivariate analysis techniques such as PCA. For PCA, three principal components with eigen values greater than 1 were extracted accounting for 75 % of total variance of the dataset (Table 2).The first principal component (PC1) accounted for 42.8 % of the total variance, followed by PC2 and PC3, which explained 18.3 % and 13.9 % of the variance, respectively.

**Table 2:** Eigen values and percentage of variance explained by the principal components.

Component	Eigenvalue	Variability (%)	Cumulative %
PC1	4.281	42.81	42.81
PC2	1.831	18.31	61.12
PC3	1.389	13.89	75.01

The loading matrix showed that the variables studied were related in different ways (Table 3). PC1 had strong positive loadings for total agricultural area, dromedary herd size, and sheep numbers. This means that this component represents the structural dimension of farms, which is mostly about farm size and livestock holdings. Farms that scored higher on PC1 had more land and more animals. Similar trends have been noted in other arid areas where the shift from nomadic to sedentary production systems has gradually altered herd structure and management practices, resulting in increased farm differentiation and variability in herd composition (Sghaier et al., 2023).

**Table 3:** Loadings of variables on the first three principal components.

Variables	Code	PC1	PC2	PC3
Total Farm Area (ha)	TFA	**0.402**	-0.299	0.335
DromedaryMortality Rate (%)	DMR	0.174	**0.780**	0.386
Reproductive Abortion Rate (%)	ABRT	-0.152	0.402	**0.704**
Total Dromedary Herd Size (head)	DHERD	**0.845**	-0.281	-0.081
Number of Dromedaries Sold (head)	DSOLD	**0.863**	-0.389	0.155
Dromedary Sale Price (TND/head)	DPRC	**0.870**	-0.367	0.149
Number of Young Sheep (head)	YSH	**0.862**	0.252	0.057
Number of Adult Sheep (head)	ASH	**0.811**	0.494	-0.089
Poultry Flock Size (head)	PFLT	0.105	0.339	**-0.694**
Sheep Sale Price (TND/head)	SPRC	**0.663**	0.430	-0.293

*(TND: The Tunisian Dinar), (ha: One hectare)

The strongest associations of PC2 were reproductive and health indicators especially dromedary mortality rate and abortion rate. As a result, this component represents the health status of the group in the production system. These results corroborate findings from several studies underscoring that improvements in herd productivity and resilience of herds in arid environments encompass strengthening the management of herd health and tailoring feeding practices to increasingly sedentary production systems (Faye, 2013; Faye, 2022).

Economic variables, especially the prices of dromedaries and sheep for sale, dominated PC3, indicating that this component reflects the economic or market-related aspects of livestock production. This economic facet highlights the increasing role of business in shaping livestock production strategies and the gradual integration of traditional pastoral systems into more market-oriented production frameworks.

**Fig. 3:**
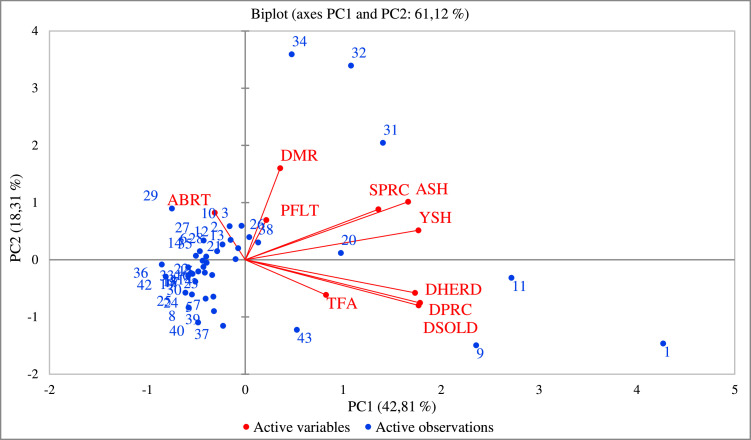
PCA biplot of camel–sheep farms based on the first two principal components (PC1 and PC2).

The graphical representation of the PCA (Fig. 3) further illustrates the relationships among variables and farms. Variables related to Market, farm structure and herd size were grouped along the positive axis of PC1, whereas reproductive indicators were mainly associated with PC2. Poultry-related variables were aligned with PC3.

Overall, the PCA results highlight that variability among camel–sheep farms is primarily driven by three major dimensions: farm structural capacity and market-related economic factors (PC1), herd health and reproductive performance (PC2), and other related livestock farms, mainly poultry (PC3).

#### 5.1.2 Identification of typological groups

To further characterize the diversity of camel–sheep farming systems and confirm the patterns revealed by the PCA, a Hierarchical Agglomerative Clustering (HAC) was performed using the coordinates of farms on the first three principal components. These components were retained because they explained 75% of the total variance, thus capturing most of the structural, health, and economic variability among farms.

**Fig. 4:**
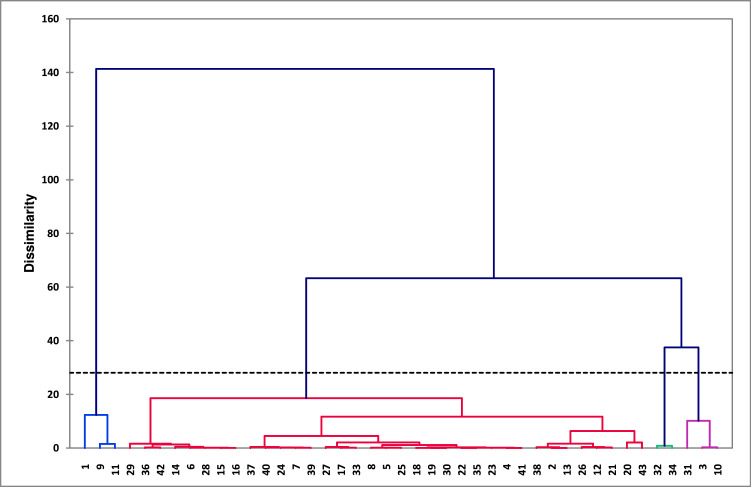
Dendrogram representing the hierarchical agglomerative clustering of camel-sheep farms using Ward method (four clusters were identified).

Ward's aggregation method was used for the clustering process, in order to minimize the within-cluster variance while maximizing differences between clusters. The Euclidean distance was used as a measure of dissimilarity between farms. The dendrogram created permitted the identification of several typological groups of farming systems. The HAC procedure indicated the presence of 4 homogeneous groups reflecting distinct types of camel–sheep farms (Fig. 4). These clusters indicate variances in herd size, production direction, and management approaches.

These groups are sorted based on important socioeconomic factors, land resources, and livestock production parameters (Table 4). This shows how different herd sizes, production directions, and herd management methods and reflect different production strategies and management constraints within the study area.

**Table 4:** Key characteristics of the typological groups derived from mean values.

Groups	Sample	AGE	TFA	ABRT	DHERD	DSOLD	ASH	GOATS	PFLT
Units	(%)	(years)	(ha)	(%)	(Head)				
Group 1	7	67	82	39	114	26	433	102	10
Group 2	81	52	36	5	21	2	57	29	9
Group3	5	68	6	16.67	13	1	445	67	15
Group4	7	60	19	36.52	31	1	225	127	80
Global means	100	54	37	22.84	28	3	113	43	14

**Group 1: Extensive Camelid-Oriented Systems**

Group 1 represents 7 % (Table 4) of the surveyed breeders and is composed of oldest farmers (mean age: 67 years) managing the largest agricultural areas (82 ha). This group is characterized by a strong specialization in camelid production, holding the highest average number of dromedaries (114 heads) and recording the highest level of dromedary sales (26 heads).

According to the survey results, these breeders are mainly located in the M’dhilla delegation, particularly in areas such as Atra, Arich, and Sagui. In addition to dromedary production, these breeders also manage substantial secondary flocks of sheep (433 heads) and goats (102 heads), reflecting a diversified agro-pastoral strategy typical of extensive production systems in arid environments.

**Group 2: Mixed Livestock Systems with Moderate Productivity Constraints**

Group 2 constitutes the dominant typological group, representing approximately 81 % (Table 4) of the surveyed farms. These breeders operate mixed livestock systems combining dromedaries, sheep, goats, and poultry.

Breeders in this group are relatively younger (mean age: 52 years) and manage medium-sized landholdings (36 ha). Their livestock numbers remain moderate across all species, with an average of 21 dromedaries, 57 sheep, and 29 goats.

**Group 3: Land-Constrained Ovine Specialists**

Group 3 represents 5 % of the surveyed breeders and is mainly located in the Redaïef and M’dhilla delegations. Farmers in this group are among the oldest respondents (mean age: 68years) and operate under severe land constraints, with an average of only 6 ha of agricultural land.

Despite limited land resources, these breeders maintain very large sheep flocks (445 heads), indicating a strong specialization in sheep production. However, this specialization is accompanied by significant production challenges, particularly a moderate abortion rate (16.67 %), which may reflect management pressures associated with high stocking density relative to available land resources.

Although dromedaries are present in these systems, their role remains secondary, with an average of 13 heads, well below the global mean of 28 heads.

**Group 4: Diversified Mixed-Species Production Systems**

Group 4 represents 7 % (Table 4) of the sample and is characterized by highly diversified livestock production strategies despite relatively limited land resources (19 ha).

Breeders in this group maintain large herds of small ruminants, including the largest goat flocks (127 heads) and substantial sheep numbers (225 heads). A distinctive feature of this group is the strong integration of poultry production, with an average of 80 birds per farm, far above the global mean (14 birds).

#### 5.1.3 Socio-economic and zootechnical profiling of breeder groups

The average age of breeders in the Gafsa region is 54.07 ± 13.30 years (Table 4), ranging from 30 to 80 years. This relatively high average age suggests limited generational renewal and reflects the declining interest of younger generations in traditional livestock production systems.

Educational attainment represents another structural constraint for the modernization of the sector. Approximately 30 % of breeders are illiterate, while 37 % and 30 % have completed primary and secondary education, respectively. Only 2 % possess a university-level education. Such low levels of formal education may limit the adoption of improved livestock management practices and technological innovations.

Land tenure patterns reveal significant disparities in land ownership. Some breeders manage relatively large agro-pastoral areas, enabling sedentary livestock systems, whereas others operate under severe land constraints, increasing their vulnerability to climatic shocks. Irrigation remains extremely limited, with only 6 % of the surveyed agricultural area benefiting from irrigation infrastructure, which further exacerbates the vulnerability of these production systems to recurrent drought conditions characteristic of southwestern Tunisia.

Animal acquisition is primarily based on inheritance (53.5 %), followed by purchases in local markets. Many breeders reported a traditional distrust of the weekly livestock markets ("souks"), due to concerns regarding uncertain animal health status. While this cautious strategy may reduce the risk of introducing infectious diseases, the heavy reliance on inherited animals may increase the risk of inbreeding and limit opportunities for genetic improvement of the Maghrebi camel population.

Livestock diversification is a widespread strategy among breeders. In addition to camel husbandry, 79 % of farmers also raise small ruminants. Specifically, 5 % of breeders raise only sheep, while 74 % maintain mixed flocks of sheep and goats.

The coexistence of multiple livestock species enhances economic flexibility and resilience, particularly under harsh climatic conditions. In this context, dromedary farming plays a strategic role as a risk-buffering asset, contributing to household income stability and resilience to drought. Recent studies have highlighted the importance of the dromedary as a key resource for pastoral resilience and market integration in arid regions of Tunisia (Faye, 2022; Sghaier et al., 2023).

Based on the typological classification, breeders can be summarized as follows:

• **Group 1:** large-scale dromedary-oriented breeders operating extensive agro-pastoral systems.
• **Group 2:** mixed livestock farmers combining several animal species in integrated production systems.
• **Group 3:** land-constrained sheep specialists with large ovine flocks
• **Group 4:** diversified breeders integrating goats and poultry production.

#### 5.1.4 Management practices and feeding strategies

The majority of dromedary production in the area that was surveyed relies on large management systems. This is because of long-standing pastoral traditions that continue even though many pastoral communities are moving from nomadic to more sedentary production systems. In these settings, dromedaries are mainly reared in low-input grazing systems, depending significantly on natural rangelands for their nutritional needs.

The dromedary's feeding habits let it eat a wide range of plants, such as shrubs, halophytic plants, and thorny plants that other domestic ruminants often avoid or can't reach (Narjisse, 1989). This adaptive feeding strategy helps the species make the most of the sparse and varied plants that are common in dry and semi-dry ecosystems. Breeders sometimes give animals locally available agricultural by-products, like cereal straw and crop residues, to supplement their diets when there isn't enough feed. This is in addition to the natural forage resources they already have.

But the way farms feed their animals is different based on things like the weather, the size of the herd, and how much money the breeders have. When there is a drought or the rangeland is badly damaged, there is less natural forage available. This means that breeders have to give their animals more extra feed to keep their herds productive and breeding well. On the other hand, when the weather is good for rain and the rangeland is more productive, most breeders rely mostly on grazing and crop by-products, which means they don't buy as much feed because it costs a lot.

In general, the way breeders feed dromedaries in the study area shows that they are flexible and can change their feeding habits based on changes in the environment, such as the availability of rangeland, changes in the weather, and economic limits.

### 5.2 Reproductive performance and health challenges

Based on our survey, herders in the Gafsa region typically engage dromedaries in reproduction at an early age, generally between 1.5 and 2 years, as a strategy to maximize overall herd productivity.

The average fertility rate of pastoral herds in the Gafsa region is remarkably high (94 %), exceeding earlier reports such as those of Faye (1997), who documented fertility rates ranging from 30 % to 35 % in extensive systems. Fertility rates across the four identified groups vary from 83.33 % to 100 % (Table 5), with Groups 1 and 3 showing significantly higher values (p < 0.05) compared to Groups 2 and 4. This difference likely reflects more effective reproductive management practices in these systems.

**Table 5:** Reproductive performance of dromedaries according to production system group.

Groups	Fertility (%)	Abortion (%)
1	100	39*
2	83.33*	5
3	100	16.67*
4	94.57*	36.52*
Means	94	22.84

* Significant difference compared with other groups within the same column (p < 0.05).

The abortion rate remains a critical constraint affecting productivity. The overall mean abortion rate is high (22.84 %), with considerable variation among groups: particularly elevated in Groups 1 and 4, moderate in Group 3 (16.67 %), and low in Group 2 (5 %). These differences suggest variability in both health status and management conditions across production systems. Infectious diseases such as brucellosis are suspected contributors, although not confirmed in this study, while nutritional deficiencies also play a major role. Undernutrition can lead to metabolic disorders, including elevated ketone bodies (e.g., beta-hydroxybutyrate), resulting in pregnancy loss.

Parasitic diseases, particularly trypanosomosis caused by *T. evansi*, represent an additional major health constraint. Serological analysis using the CATT/*T. evansi* test revealed a very high prevalence (90.91 %) among sampled animals, indicating widespread infection in the study area. Seroprevalence reached 100 % in North Gafsa, 93.33 % in M’dhilla, and 84.62 % in South Gafsa (Table 6), highlighting the endemic nature of the disease. This high prevalence is consistent with clinical signs reported by breeders, including weight loss, reduced feed intake, fever, locomotor disorders, and lymph node enlargement.

**Table 6:** Seroprevalence of *Trypanosoma evansi* infection in sampled dromedaries across the delegations of the Gafsa region.

Delegation	Number of breeders	Total herd size	Number of samples	Percentage of positive tests(%)
Mdhilla	6	340	30	93.33
Gafsa South	9	167	13	84.62
Gafsa North	1	16	1	100

The widespread occurrence of *T. evansi* can be explained by the extensive pastoral systems practiced in the region, characterized by communal grazing and shared watering points, which favor exposure to mechanical vectors such as Tabanus and Stomoxys flies. Similar epidemiological patterns have been reported in other arid and semi-arid regions of North Africa (Kalthoum et al., 2021; Gharbi et al., 2025). *Trypanosomosis* is known to weaken animals and contribute to reproductive disorders, including increased abortion rates and reduced overall productivity (Darwish et al., 2023). However, the cross-sectional design of the present study provides only a snapshot of the epidemiological situation, and temporal variations related to vector dynamics or treatment practices should be considered (Selim et al., 2022).

In addition to reproductive disorders, mortality represents a major constraint for herd productivity. The average adult mortality rate is 4.81 % ± 10.83, while calf mortality reaches 10.51 % ± 14.10 (Table 7), which is considerably higher than values reported in other North African contexts (Siboukeur, 2007). Mortality rates vary significantly among groups, with particularly high calf mortality observed in Group 1 (35 %).

**Table 7:** Mortality rates of adult dromedaries and young calves by production system groups.

Groups	Adult dromedary mortality (%)	Young calf mortality (%)
1	1.5	35*
2	2	9.33
3	2.33	12.67*
4	5.46*	9.03
Means	4.81	10.51

* Significant difference compared with other groups within the same column (p < 0.05).

When reproductive and mortality indicators are considered together, clear differences emerge among production systems. Although Groups 1 and 3 exhibit high fertility rates, Group 1 is characterized by elevated abortion and calf mortality rates, ultimately reducing effective herd productivity. In contrast, Group 2 shows a more balanced profile, combining lower abortion rates and moderate mortality, suggesting greater system stability. These findings underline that herd performance depends not only on fertility but also on reproductive losses and juvenile survival.

The main causes of mortality reported by breeders include diarrheal diseases and enterotoxemia, along with parasitic infestations (ticks, mange, and trypanosomosis), malnutrition, and intoxication. Environmental factors also play a role, particularly pollution associated with phosphate mining activities in the Gafsa region, which may contaminate water resources and affect animal health.

Overall, these results confirm that health constraints—particularly parasitic diseases such as *T. evansi,* nutritional deficiencies, and infectious conditions—constitute major limiting factors for the productivity and sustainability of dromedary production systems in southwestern Tunisia.

## 6 Conclusion

The present study provides a comprehensive characterization of dromedary breeding systems in the Gafsa region, highlighting a sector dominated by traditional extensive management practices and generally low levels of productivity. Dromedary farming is largely integrated with small ruminant production systems, particularly sheep and goats, and remains primarily oriented toward meat production while being strongly embedded in local socio-cultural traditions.

The surveyed herds face substantial health constraints, notably high prevalences of ectoparasitic infestations, including ticks and mange, and haemoparasitic infections such as trypanosomosis. These sanitary challenges are associated with considerable productive losses, reflected by elevated rates of juvenile mortality and abortion. Such indicators point to significant weaknesses in herd health management.

Overall, the findings reveal the structural fragility of dromedary farming systems in the region, driven mainly by chronic nutritional deficiencies and limited technical expertise among breeders. In many cases, management practices are guided by tradition rather than by adaptive strategies aligned with environmental constraints and productivity objectives.

Improving the performance and sustainability of the dromedary sector in Gafsa requires targeted and coordinated interventions. Priority actions should include strengthening farmers’ technical capacities through training and extension services, promoting improved feeding strategies, and enhancing animal health management through regular vaccination and disease surveillance programs. In parallel, encouraging the involvement of younger, more technically trained stakeholders could play a key role in modernizing production systems and ensuring the long-term viability of dromedary farming in this arid environment.

## Funding statement

This research received no specific grant from any funding agency in the public, commercial, or not-for-profit sectors.

## CRediT authorship contribution statement

**Raoudha Sadraoui:** Writing – original draft, Validation, Software, Methodology, Data curation, Conceptualization. **Sana Khaldi:** Writing – original draft, Visualization, Methodology. **Imen Kallel:** Software, Formal analysis. **Mohamed Jaouad:** Validation, Supervision.

## Declaration of competing interest

The authors declare that they have no known competing financial interests or personal relationships that could have appeared to influence the work reported in this paper.

## Appendix Supplementary materials

Image, application 1
