# Supplementary material for: Practices, management, and typology of dromedary livestock systems and health constraints in southwestern Tunisia: The case of the Gafsa region
Source: Vet Anim Sci. 2026 Apr 1;32:100646. doi: 10.1016/j.vas.2026.100646 (PMC13091137; doi:10.1016/j.vas.2026.100646)
Supplement: Supplementary file 1 [file mmc1.docx]

**Supplementary File S1**

# **Supplementary File S1. Survey Questionnaire for Camel Breeding Systems**

**General Information**

Survey Date: .....................................................
Locality (specific site): .....................................................
Imada (administrative district): .....................................................
Delegation: .....................................................
Governorate: .....................................................

Nearest village: .....................................................
Distance to the village (km): .....................................................

**Farmer Identification**

Name of the farm owner/household head: .....................................................
Age: .....................................................

Place of residence: .....................................................
Distance between residence and farm (km): .....................................................

Educationallevel:

- Illiterate / Quranicschool (Koutteb)
- Primaryeducation
- Secondaryeducation
- Highereducation

Main economic activity: .....................................................
Secondary economic activity: .....................................................

Primary agricultural activity: .....................................................

**Land Tenure and Farm Structure**

Total Agricultural Area (TAA) (ha): .....................................................
Utilized Agricultural Area (UAA) (ha): .....................................................
Uncultivated land (ha): .....................................................

Irrigated area (ha): .....................................................
Water source: .....................................................

Land Tenure Status

- Owned land (%) .....................................................
- Rented land (%) .....................................................
- Other (specify): .....................................................

**Farm Management**

Farm management is ensured by:

- The farmerhimself
- A familymember
- Other (specify): .....................................................

**Crop Activities and Land Use**

1. What is the total land area of the farm?

| Total Area (ha) | Cultivated Area (ha) | Uncultivated Area (ha) | Irrigated Area (ha) |
| --- | --- | --- | --- |

**Herd Composition**

1. How many animals do you currently own, and what is the composition of your herd?

| Animal Species | Category | Current Number | Association In (TLU) | Association Out (TLU) |
| --- | --- | --- | --- | --- |
| Camels | Female calves (<1 year) |  |  |  |
| Camels | Male calves (<1 year) |  |  |  |
| Camels | Subadultfemales (1–4 years) |  |  |  |
| Camels | Subadult males (1–4 years) |  |  |  |
| Camels | Adultfemales (>4 years) |  |  |  |
| Camels | Adult males (>4 years) |  |  |  |
| Sheep | Young (<6 months) |  |  |  |
| Sheep | Adults |  |  |  |
| Goats | Young (<6 months) |  |  |  |
| Goats | Adults |  |  |  |
| Other | Horses |  |  |  |
| Other | Mules/Donkeys |  |  |  |
| Other | Poultry |  |  |  |
| Other | Beehives |  |  |  |

**Animal Breeds**

**2. Which breeds are present in the herd?**

Camel breeds: __________________________
Sheep breeds: __________________________
Goat breeds: __________________________

**Origin of Animals**

**Replacement rate**

Males: .....................................................
Females: .....................................................

**Origin of replacement animals**

Males: .....................................................
Females: .....................................................

**Selection criteria for replacement animals**

............................................................................................

**Culling Management**

**Culling rate:** .....................................................
**Age at culling:** .....................................................

**Main cullingcriteria**

- Low milk production
- Body conformation
- Abortion history
- Fertilityproblems
- Maternalability
- Age
- Severe diseases
- Other (specify): .....................................................

**Reproductive Management**

**Breeding season**

Start: .....................................................
End: .....................................................

**Number of females per male:** .....................................................

**Calving period**

Start: .....................................................
End: .....................................................

**Health and Mortality**

**Causes of abortion:**
............................................................................................

**Mortality rate**

Young animals: .....................................................
Adult animals: .....................................................

**Main causes of mortality**

Young animals:
............................................................................................

Adult animals:
............................................................................................

**Weaning and Fattening**

**Start of weaning:** .....................................................
**Average weaning age:** .....................................................

**Fattening practiced:** Yes / No
**Fattening period:** .....................................................

**Herd Mobility**

**1. What was the mobility pattern of the camel herd and small ruminant herd during the last 12 months?**

| **Month** | **Type of Grazing Area** | **Location** | **Distance (km)** | **% Camels** | **% Small Ruminants** | **Herding Responsibility** |
| --- | --- | --- | --- | --- | --- | --- |

Types of grazingareas:

1. Private rangeland
2. Collective rangeland
3. Grazingoutside the village territory
4. Other (specify)

Herding responsibility:

1. Farmer himself
2. Son
3. Both
4. Other (specify)

**Herd Size Evolution**

Over the last five years, what were the total camel herd sizes and the number of months animals remained sedentary?

| **Year** | **Total Herd Size** | **Number of MonthsStationary** |
| --- | --- | --- |
| May 2016 – May 2017 |  |  |
| May 2015 – May 2016 |  |  |
| May 2014 – May 2015 |  |  |
| May 2013 – May 2014 |  |  |
| May 2012 – May 2013 |  |  |

**Animal Flows**

**Animal Transactions During the Last 12 Months**

| Animal  Species | Category | Purchases (Number/Value) | Sales (Number/Value) | Self‑consumption (Number/Value) | Gifts/Barter (Received/Given) |
| --- | --- | --- | --- | --- | --- |
| Camels | Female calves (<1 year) |  |  |  |  |
| Camels | Male calves (<1 year) |  |  |  |  |
| Camels | Subadultfemales (1–4 years) |  |  |  |  |
| Camels | Subadult males (1–4 years) |  |  |  |  |
| Camels | Adultfemales (>4 years) |  |  |  |  |
| Camels | Adult males (>4 years) |  |  |  |  |
| Sheep | Young (<6 months) |  |  |  |  |
| Sheep | Adults |  |  |  |  |
| Goats | Young (<6 months) |  |  |  |  |
| Goats | Adults |  |  |  |  |
